# A comprehensive grid to evaluate case management’s expected effectiveness for community-dwelling frail older people: results from a multiple, embedded case study

**DOI:** 10.1186/s12877-015-0069-1

**Published:** 2015-06-18

**Authors:** Thérèse Van Durme, Olivier Schmitz, Sophie Cès, Sibyl Anthierens, Patrick Maggi, Sam Delye, Johanna De Almeida Mello, Anja Declercq, Jean Macq, Roy Remmen, Isabelle Aujoulat

**Affiliations:** IRSS, Institute of Health and Society, Université catholique de Louvain clos Chapelle-aux-Champs, 30.13 B-1200 Brussels, Belgium; Faculty of Medicine and Health Care Sciences, Universiteit Antwerpen Universiteitsplein, 1 B-2610 Wilrijk, Belgium; Faculty of Public Health Université de Liège Avenue de l’hôpital, 3 B-4000 Liège, Belgium; LUCAS, Centre for Care Research and Consultancy KU Leuven (University of Leuven), Kapucijnenvoer 39, B-3000 Leuven, Belgium

**Keywords:** Case management, Frail elderly, Programme evaluation

## Abstract

**Background:**

Case management is a type of intervention expected to improve the quality of care and therefore the quality of life of frail, community-dwelling older people while delaying institutionalisation in nursing homes. However, the heterogeneity, multidimensionality and complexity of these interventions make their evaluation by the means of classical approaches inadequate. Our objective was twofold: (i) to propose a tool allowing for the identification of the key components that explain the success of case management for this population and (ii) to propose a typology based on the results of this tool.

**Methods:**

The process started with a multiple embedded case study design in order to identify the key components of case management. Based on the results of this first step, data were collected among 22 case management interventions, in order to evaluate their expected effectiveness. Finally, multiple correspondence analyses was conducted to propose a typology of case management. The overall approach was informed by Wagner’s Chronic Care Model and the theory of complexity.

**Results:**

The study identified a total of 23 interacting key components. Based on the clustering of response patterns of the 22 case management projects included in our study, three types of case management programmes were evidenced, situated on a continuum from a more “socially-oriented” type towards a more “clinically-oriented” type of case management. The type of feedback provided to the general practitioner about both the global geriatric assessment and the result of the intervention turned out to be the most discriminant component between the types.

**Conclusion:**

The study design allowed to produce a tool that can be used to distinguish between different types of case management interventions and further evaluate their effect on frail older people in terms of the delaying institutionalisation, functional and cognitive status, quality of life and societal costs.

**Electronic supplementary material:**

The online version of this article (doi:10.1186/s12877-015-0069-1) contains supplementary material, which is available to authorized users.

## Background

As in many high-income countries, the Belgian healthcare system is considered to be complex for people who need long term care [1]. Frail older people often suffer from multiple, interacting morbidities and incapacities. They therefore need care of different providers, from both the health care and the social care sector. The health care system, supposedly capable of providing an adequate answer to these peoples’ needs is in fact (a) mainly driven by a logic of acute care, while these people need chronic care [2]; (b) single-disease-centred, while the majority of these people have at least two or more chronic conditions [3, 4]; (c) hospital-centred, while most of them still live at home [5]; and (d) characterized by a poor level of organisation at the primary care level [6]. The risk of worsening of this situation in Belgium is high. Indeed, the split and evolving decision-making power between different policy levels for connected issues lead to a high probability that fragmented care is delivered [1]. This means that the information about the available care agencies and reimbursement statuses is scattered around different levels, changing all the time and very confusing for the care providers, and all the more for the older people and their families.

Navigating through such a complex health care landscape, in order to get the appropriate (health) care can be very difficult. Moreover, once the care providers have been identified by the beneficiaries, their intervention needs to be integrated, so that it is not overlapping and that one care provider knows and realizes what the other care provider does [6]. Wagner’s Chronic Care Model (CCM) is believed to provide a framework to restructure the health system towards integrated, proactive, consistent and continuous care, and thus, anticipate some acute exacerbations or lessen their consequences. Six interacting elements are essential in the CCM: the links with the community, the health system, self-management support, tailored delivery system design, help for decision support and adequate clinical information systems [2, 6, 7].

Case management for people with complex care needs could be one of the effective strategies within that framework [8, 9]. Namely, case management is expected to support the provision of integrated care. It is “a collaborative process of assessment, planning, facilitation, care coordination, evaluation, and advocacy for options and services to meet an individual’s and family’s comprehensive health needs through communication and available resources to promote quality, cost-effective outcomes” (Case Management Society of America, 2010) [10].

In 2009 the Belgian National Institute of Healthcare and Disability Insurance (NIHDI) launched a nationwide call to generate innovative forms of care, to enhance the capacities of frail older people to remain at home if they wanted to [11]. Based on the submission files describing how and with which means they planned to delay the institutionalisation of older people, the projects were selected by a jury and, if accepted, received funding during four years (2010–2014). Project conceivers were given very little directions as to how and with which means they were to organise their interventions, except for the inclusion criteria of the beneficiaries (frail older people, defined below) and to have a nursing home or coordination centre as partner, as well as to use a web-based comprehensive geriatric assessment (InterRAI-HC) [12]. They may therefore be considered bottom-up designed projects.

Moreover, to evaluate in how far the different projects contributed to delaying institutionalisation, the NIHDI asked a consortium of universities to evaluate the effectiveness of the projects to delay definitive institutionalisation in nursing homes, to maintain or improve the functional status and quality of life while assessing their cost, for the NIHDI, the older people, and the impact on the burden of their main informal caregiver.

In total, 67 projects were approved by the NIHDI, of which 22 were projects with a focus on case management. These projects met at least four of six of the elements of the Case Management Society of America’s (CMSA) definition of case management [10]. However, as this intervention is new in Belgium, no references or guidelines exist in Belgium about who should take on this role and which functions should be carried out to achieve positive outcomes.

Besides being complex interventions, the 22 case management projects were characterised by a high level of diversity. Not only because they were (a) bottom-up designed projects but also (b) despite including only frail older beneficiaries (attested by either a score on the Edmonton Frail Scale [13] of 6 or more or having a diagnosis of dementia), the manifestation of frailty could be very diverse. Moreover, (c) because there are currently no standards of practice for case management available in Belgium, the case management programmes differed in *size* (number of professionals involved and caseload), in *location* (French speaking, Dutch speaking, German speaking regions of Belgium, each presenting different policies possibly influencing case management implementation), in the *profile of the case manager* (nurses or non-nurses, sometimes even nursing assistants), the *degree of involvement* of the primary care agencies (among which the general practitioner (GP)), the *use of the results of the comprehensive geriatric assessment*, i.e. the InterRAI Home Care instrument (HC), that was a cornerstone in the projects [12] and in *time* spent on case management.

The evaluation of this type of complex intervention, affecting possibly the health system in which it is implemented alls for mixed-methods approaches [14]. Therefore, the evaluation of these case management projects followed a triangulation process in order to build a comprehensive perspective including: [1] the description of the projects as a set of interventions aiming at improving outcomes for frail older people and their informal caregivers; [2] the evaluation of the (statistical) association between a given type of population, intervention, outcome and cost; [3] the analysis of the implementation process as a way to identify the mechanisms and conditions that underlie project effectiveness in a given context. The main outcomes under study were the delay of permanent institutionalisation, maintaining or improving physical functioning (ADL [15], IADL [16]), cognitive functioning (CPS [17]), depressive status (DRS [18]), quality of life [19] and the informal caregivers’ perceived burden [20].The overall design of the evaluation is detailed elsewhere [21].

Alongside evaluating the effectiveness of these interventions the process started by “opening the black box” and a thorough look at the components and implementation processes of the 22 case management projects in their local contexts. In order to standardize the description of the various implementation processes as well as the various projects’ structural characteristics, a normative grid was needed. This grid was to be used to capture what the stakeholders of the projects believed to be key components explaining the successes or failures of their projects and the likely positive outcomes for the beneficiaries. This paper describes the empirical, theory-driven elaboration of this grid. The grid is then used to evaluate 22 case management projects whose score patterns on the grid lead to a proposal of a typology of case management interventions. The results of the performance of these programmes are presented in the full report [22].

## Methods

### A. Elaborating the grid-multiple, embedded case studies over a four-year period (2010–2014)

The evaluation process started with multiple, embedded case studies of six projects [23]. The three aims of the case studies were: [1] to provide a precise and narrative description of the project components over time (structure and process factors) that would likely lead to positive outcomes; [2] to identify the contextual factors that played a role during the implementation process of the projects and the way they adapted to changes (i.e. *external*, like changes in partnership, or *internal*, like turnover of professionals etc.); [3] to build an overall analytical framework to explain the success or failure of a project, from a normative point of view [24]. The term normative refers here to assumptions and expectations about what should happen if the components of the projects were present [25] . As the relation between the type of projects and the outcome for frail older people was initially unknown, cases were selected to obtain as much diversity as possible regarding the projects’ characteristics [26, 27]. These features included the profile of the case manager, the geographic location, the size of the project (staff or intended caseload), the definition of the target population, the number of different disciplines, the type and size of the partnership, etc. This diversity increased the credibility concerning the richness of the contextual and structural variables taken into account in the study. The multiple case studies were performed by a multidisciplinary team of five researchers with a background in public health, sociology, nursing and occupational therapy (OS, MLH, PM, SD & TVD).

- Data collection

Typically, case studies combine multiple sources of qualitative and quantitative data, used in a complementary way [23, 27]. The following sources of qualitative data were used: [1] project submission files, in which projects described how they intended to support the maintaining at home of the frail older people, including means (staff and equipment) and partners, etc.; [2] annual semi-structured interviews with the stakeholders working in the projects (coordinators, persons responsible for the implementation of the project and frontline workers); [3] yearly questionnaires containing open-ended questions collecting data about the organisational functioning and about adaptations in the components of their projects and [4] written documentation, such as advertising flyers, projects website, etc. Researchers took the extra precaution to provide feed-back of their own understanding of the data, in order to validate the results with the stakeholders of the projects. The use of the data for this study was approved by the Ethics Committee of the Université catholique de Louvain under the reference B40320108337.

(b) Audit trail and data analysis

Data of the six case studies were then analysed to allow for explanation building and the proposition of logical models, representing the logic of the intervention, or “programme theory”. A logic of an intervention demonstrates how an intervention is expected to contribute to possible or actual impacts, which can be either positive or negative [28]. As such the questions were: What are the objectives of the projects? Which activities are implemented to reach these objectives? Which mechanisms can explain a potential impact on the frail older people or their informal caregivers’ outcomes? This information drew upon Ridde and Haddad’s article about pragmatic evaluations of complex interventions [29].

Templates were used for the organisation of the data. They allowed for the standardised collection of data from the six cases by the five researchers involved in the process. Further, they allowed constant comparison within and between cases. Moreover, the method aimed at creating a case study database using multiple sources of evidence, thus allowing the analysis and a chain of evidence to be maintained [23]. Specific attention was paid to emerging themes or main “lessons learned”. They dealt with the implementation process of the project, including the description of the differences between the “model” of the project as planned and the project as implemented in practice.

The following step included a between-cases analysis of the projects, trying to identify the essential components of the projects and of the context in [1] achieving their implementation and [2] being successful in reaching the desired outcomes for the older people. These desired outcomes aimed at by the projects could be so-called “hard” outcomes (i.e. delaying institutionalization, improving functional status and health, alleviating the burden of their informal caregivers, etc.) but also “soft” outcomes, such as enhancing coping skills, access to adequate information and service options. In other words, if desirable outcomes were to be achieved, what would be the essential components of their projects likely to explain these successes?

#### Content of the normative grid

**Fig. 1:**
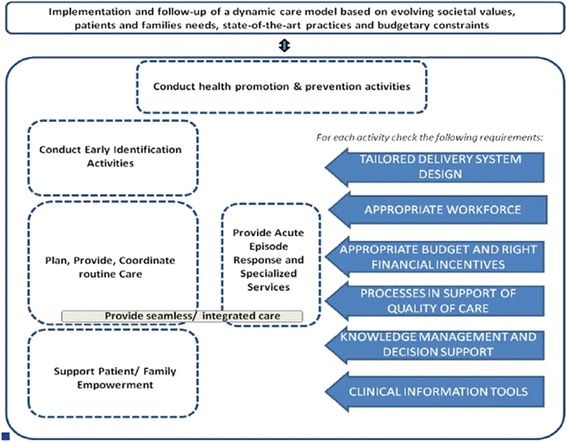
Wagner’s Chronic Care Model, adapted to the Belgian context [5]

The process resulted in a list of 23 components, which were then displayed along with Wagner’s Chronic Care Model [30]. The CCM model adapted to the Belgian context by the KCE [6] was used. The main difference with the original model lies in the fact that the model has been operationalised into activities and requirements that are needed to achieve the effectiveness of the Chronic Care Model (arrows in blue in Fig. 1). Domains of the original model and the requirements of the KCE model deemed to be relevant for the evaluation of case management projects were selected after discussion among researchers. These results were validated by the stakeholders of the 16 projects which were not part of the case studies during yearly focus groups.

#### Transformation of qualitative data into quantitative data

In order to be able to evaluate the 22 case management projects, a maximum of four levels of was provided for each of the 23 components. They ranged from 0 (the lowest level of achievement) to 3 (highest level of achievement). All the components and the relevance of all criteria were discussed within the consortium of researchers, based on the results of the multiple case studies. The complete list of components, each with their criteria justifying a given score, is displayed in the Additional file 1. Each of the 22 case management projects (including those of the case studies) was then screened by at least two researchers independently and each component was given a score. When diversity occurred within an organisation, e.g. when some professionals in an organisation were skilled and others not, the lowest score was provided. Results were then compared and discussed until consensus was reached.

### B. Testing the grid-analysis of multiple correspondences

#### Searching for response patterns on the normative grid

The grid with the 23 components was then tested by the means of a factorial analysis of multiple correspondences (MCA) using STATA 11® among the 22 case management projects. This technique can be seen as a type of principal component analysis applied to a contingency table. It is an exploratory analysis and allows for the study of the statistical association between several qualitative or categorical variables, in order to show nonlinear relations between those variables [31, 32]. MCA explores the relationships among a set of multiple variables in a table by decomposing the deviation from independence of this table (i.e. independence of the variables of the columns against the rows of the table). By default, STATA uses Burt’s table, i.e. a symmetric matrix of all two-way cross-tabulations between the categorical variables to display all the variables. When performing a correspondence analysis of a table, it is possible to make a graph in which each point represents either a variable of a row or of a column. The aim is to seek factorial axes, i.e. the axes showing the largest deformation (= inertia) of the cloud of points, in order to reduce the dimensions of the table [31]. To define the number of dimensions to include in the analysis, two criteria were retained; firstly a scree test; secondly, eigenvalues higher than the mean eigenvalue, in this case 1/23 = 0.04, as recommended by Benzécri [33]. Other authors however [32, 34] recommend two-dimensional pictures of data, which facilitates data interpretation and this is what we did. A next step was then to try to show the quality of the representation and the relative inertia of each of the modalities of the two main first dimensions. For this, the weight of each modality and its contribution to the total inertia of the cloud of points was searched. Only the modalities showing the best quality of the representation of the factorial axes and those which were the most contributive were retained (i.e. ≥ 0.04) [32].

#### Searching for a typology of case management projects

Additionally, even if MCA is mainly used as an exploratory technique, it can be a powerful tool because it may show groupings of categories of variables in the dimensional spaces, providing important insights on relationships between categories or, in other words, multivariate treatment of the data through simultaneous consideration of multiple categorical variables [34] . By doing so, it was then possible to identify which of the scores of the 23 project components showed an attraction or repulsion to each other and build a typology of case management projects. This was done by clustering the modalities of variables which were very closely situated in the graph on the two first dimensions. Because these were composite scores of the variables, the multidimensionality of the variables was preserved when clustering the responses.

#### Grouping case management projects

In a further stage, the numbers of projects sharing similar response modalities were calculated. A satisfactory approximation of a type was provided if projects were grouped that shared at least the half of the maximum response modalities of their type [32]. Once the typology was done, so-called supplementary variables were added, such as the profile of the case manager, the geographic location, etc. This avoided a projection of the typology of projects from a priori ideas [31]. Because STATA 11® does not allow showing the labels in the graph, Trideux® software was used to display the results of the MCA [35]. The significance level for the deviation from independence was set at 5 %.

## Results

### A. Basic description of the case management projects

The 22 case management projects showed some similarities, as all case managers in the projects were working as a team. The teams consisted of (a) only nurse case managers (*n =* 7; i.e. 33.3 %); (b) both nurses and social workers (*n* = 8; i.e. 38.1 %); (c) both nurses and occupational therapists (*n* = 1; 4.7 %) or social workers and other professionals (i.e. psychologists or occupational therapists; *n* = 5 or 23.8 %). Beneficiaries could be referred to the projects through different channels: their informal caregiver, home care services, general practitioners, social services of hospitals, community nurses, etc. They could be included in the project if they met the inclusion criteria set by the Royal Decree. Differences between case management projects were related, amongst others, to the mean number of patients in active file in the projects, i.e. 98, ranging from 4 to 189 and the duration of case management from 15 days to 36 months (mean = 6 months). Sixteen case management projects were situated in the Flemish region, three in the Walloon region, two in the Brussels region and one in the German-speaking region. Each of these regions has separate decision powers and funding systems regarding social care, possibly impacting the embeddedness of case management projects.

### Presentation of the normative grid with the key components of the projects

Based on the data of the multiple case studies, and reflecting the perspective of the stakeholders involved in the projects, two domains and six requirements were identified as the most relevant explaining the success of and the implementation of the case management interventions: the appropriateness of the workforce, the tailored service design and organisation, the self-management and support, the community linkages, the appropriate financial incentives, the processes in support of the quality of care, the knowledge management and decision-support and the clinical information tools.

As expected, stakeholders of the projects also reported the importance of “soft” outcomes, besides the “hard” outcomes asked for by the NIHDI. Amongst these, the projects identified nine desired outcomes of case management for frail older people, such as (a) improved health and healthcare literacy (including about the project’s functioning); (b) older people’s, informal and professional caregiver’s satisfaction with care; (c) care providers and frail older peoples’ facilitated access to the relevant information; (d) better detection and anticipation of crisis situations; (e) lower threshold for help-seeking behaviours; (f) increased sense of security; (g) decreased sense of social isolation; (h) increased sense of belonging to a community and (i) increased coping abilities and sense of control.

The overall table with 23 components displayed into eight domains or requirements of the CCM, along with an operational definition and criteria used to assess the level of achievement of each component within these domains are provided in the Additional file 1.

- The appropriateness of the workforce

Adequate workforce emerged as an important requirement in order for the project to achieve desired results, i.e. the recruitment of skilled and trained professionals, with either an expertise in geriatric care or a specific knowledge about the local resources. This was stressed by a project manager as follows: *“An important added value to make the case management succeed is the recruitment of a geriatric nurse with a 10-year experience in liaison function in a hospital”* (interview1 project conceiver, 2010). Moreover, as the case manager is to be the reference person for the frail older person, a low turnover rate of case managers was seen as a pivotal component. This meant that whenever this reference person was on sick leave and had to be temporarily replaced, projects favoured experience over a specific profile (e.g. they preferred to recruit an experienced occupational therapist as a case manager instead of a newly trained community nurse). However, the preference was for a community nurse, who is supposed to [1] be trained to care for frail older people in a holistic perspective and [2] have a good knowledge of the resources within the local system, including a clear view of other (health and social) care providers’ roles.

(b) Tailored service design and organisation

The case management projects under study were pilot projects. Their funding therefore depended on the achievement of their expected caseload as described in the submission files. Therefore, the achievement of the caseload was seen as an important component that was closely linked to the adequacy of their inclusion or exclusion criteria. For example, some projects expanded their recruitment area in order to be able to achieve their caseload without changing their clinical inclusion criteria. Other projects adapted their inclusion or exclusion criteria to the demands of the potential clients and referrers. Indeed, as the innovative project grew in maturity, the awareness for inclusion criteria allowing identifying older people to whom the case management would benefit the most, increased. Finally, the decision process regarding the internal organisation of the project itself should be shared with all the actors of the project, because this was seen as a safeguard for the adequacy of the tailored service design and organisation (e.g. related to the adequacy of the inclusion criteria, as described above). This was highlighted by a nurse case manager: *“A year after the start of the project, our team decided to restrict its inclusion criteria because we observed that the intervention proposed did not lead to satisfactory results in people with early stage dementia or psychosis”.* (Interview3 case manager, 2012)

(c) Self-management and support

Self-management support was mentioned as an important feature of the process. This was approximated by the degree to which the concerns of the older people and their informal caregivers were taken into account in the care planning and the degree to which they were involved in multidisciplinary meetings.

(d) Community linkages

The existence of a structural link with organisations that could refer beneficiaries was seen as important. Indeed, this enabled the case management project to achieve their caseload and to include beneficiaries who were most likely to benefit from the case management intervention. Moreover, this facilitated the knowledge of the mutual role definition of the professionals inside these organisations. As was stated by a project manager: *“If we did not have these links, we wouldn’t even have achieved a caseload of 80 (instead of 150). Even if there is a difference among partners, the referral process can only be effective if the partner organisations know precisely what you are doing, of course.”* (Interview3 project manager, 2012). The adequate referral to the project facilitated in turn the referral from the case manager to the services as needed by the older people, based on the comprehensive geriatric assessment. This was observed through formal partnerships with coordination (home nursing agencies) and community agencies (Public Centres for Social Welfare).

(e) The appropriate financial incentives

In order to benefit from the intervention, the care had to be affordable for the beneficiary. On the one hand, if the case management intervention was free for the beneficiaries, the additional services provided by the projects (i.e. psychological support by a psychologist or nursing care provided during the night) were still to be paid by the older people. As a social worker case manager stated: *“Now, a pension in Belgium is not terribly high. We observe this general trend in home care: people ask to pare the number of care hours. They say: “You’ll only have to come once per week.” Or “Come one hour less.” I don’t think this is specific for any type of care.”* (Interview3 case manager, 2012). On the other hand, inadequate financing of the project, because of over-or more frequently, underestimation of the financial cost of staff, could lead to having to accept higher workloads. This would result in pressure on the time spent in case management. Furthermore, in the same dimension, financial incentives for general practitioner’s participation was also seen as important, as they are crucial partners in case management. However in the Belgian mainly fee-for-service system, participating in coordination activities is not adequately financed.

(f) Processes in support of quality of care

The use of quality or performance indicators was seen as important to monitor both the implementation process and the effectiveness of the project. In most projects, coordinators planned patients’ satisfaction surveys; along with performance indicators (e.g. number of services delivered per older person, according to their status of dependence). At the beneficiaries’ level, the monitoring of the care plan was seen as crucial to make sure the current organisation of the care was still adequate and this monitoring should be structurally planned. At the same time, provision of feed-back about the condition of the frail older people to their general practitioner was in line with this rationale. However, this was not always achieved: *“At each new inclusion of a patient the case manager goes to the GP’s surgery, in order to fill out together the medical part of the InterRAI-HC. However, the collaboration with the patient’s GP remains limited; the only « feed-back » we provide occur during coordination meetings.*” (Project questionnaire, 2012).

(g) Knowledge management and decision-support

Stakeholders of the projects reported that the use of results of research was important to foster high quality care, ideally also leading to the use of evidence-based protocols or guidelines, and when possible shared with professionals outside the project organisation. This was reported through a project questionnaire: *“What we find really useful is to be able to use the results of the InterRAI-HC instrument, a validated tool which enables us to assess the situation of the person by a multidisciplinary team, really centered on the beneficiary, which in turn improves his engagement in the planning of his own care. Moreover, blind spots in care needs are made visible and during multidisciplinary meetings there are fewer discrepancies regarding problem situations to be discussed. There is a common language, which also improves the discussion about the beneficiary and fosters better quality of the care. There is a sense of continuity of the care, a structural support”.* (Project questionnaire, 2013). Reflective discussions among peers or planned supervisions and multidisciplinary group meetings were viewed as important to enhance the knowledge of case managers.

(h) Clinical information tools

The presence and use of an electronic patient record and a registry, including a list of beneficiaries of the projects and reminders to providers to plan care were important facilitators of the process. This was illustrated by a project in their initial submission file: “*Our registry can be seen as the brain of the organisation of the care. It manages the whole activity of the outreach team. All members of the project encode their appointments with beneficiaries and it is accessible through VPN*”. (Submission file, 2010).

### B. Use of the normative grid with the key components of the projects

#### Finding response patterns on the normative grid

Two dimensions were retained for the MCA. The first and second dimensions presented had respectively an eigenvalue of 0.087802 (percentage of total = 28.9 %) and 0.062654 (percentage of total = 21.4 %). The principal inertia was rather low, i.e. 0.12 and 0.056 for the two first dimensions, predicting a poor level of explanation (i.e. 54.89 %) of the total inertia. Only the modalities showing the best quality of the representation of the factorial axes and those which were the most contributive were retained (i.e. ≥ 0.04; [32]). They are shown in Table 1.

**Table 1:** Most contributive modalities to the factorial axes, displayed along the domains of the Chronic Care Model [5]

Domains of the Chronic Care Model	Criteria	Abbreviation
Appropriate workforce	Turnover of the case manager	tur
	Skills of the case managers	ski
Tailored service design and organisation	Adequacy of the inclusion criteria	ade
Community linkages	Partnership with coordination centres	par
Appropriate financial incentives	Financial accessibility to the programme	fin
	Financial incentives to engage the general practitioner	inc
Processes in support of quality of care	Feed-back to the general practitioner	fba
	Monitoring of the care plan	mon
Knowledge management and decision-support	Use of evidence-based, multidisciplinary protocols	ebp
	Presence of reflective discussion among peers	int
Clinical information tools	Use of a registry	reg
	Reminders and prompts to organise the care	rem

#### Coming to a typology of case management projects

**Fig. 2:**
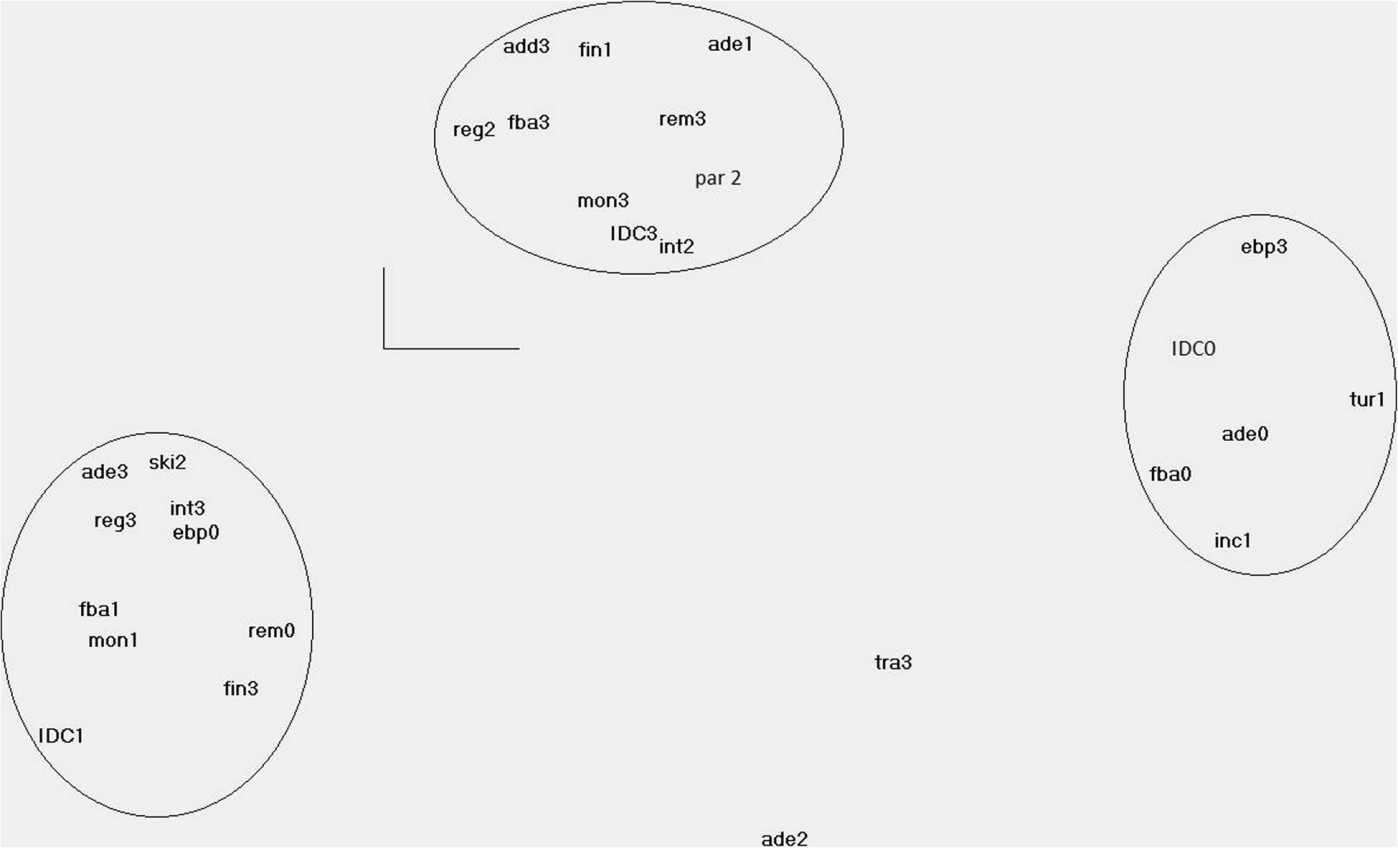
Multiple correspondence analysis (MCA) of the responses of the normative grid of case management projects. The responses are grouped around the most contributive responses. Scores range from 0 to 3. The higher the score, the higher the level of achievement. ade = “Adequacy of the inclusion criteria”; ebp = “Use of evidence-based, multidisciplinary protocols”; fba = “Feed-back to the general practitioner”; fin = “Financial accessibility to the programme”; inc = “Financial incentives to engage the general practitioner”; int = “Presence of reflective discussion groups among peers”; mon = “Monitoring of the care plan”; par = “Partnership with coordination centres”; reg = “Use of a registry”; rem = “Reminders and prompts to organise the care”; ski = “Skills of the case managers”; tur = “Turnover of the case manager”

The modalities of the variables are represented on a graph in function of their coordinates on the two main first dimensions. The X-axis represents the first dimension and the Y-axis the second one. On each of the axes it is then possible to view the modalities of which the contribution to the formation of the axis is higher than the mean. In other words, this representation allows viewing the attractivity of the responses within a dimension and their repulsion (or opposition) between these two responses. The threshold was augmented until the single most contributive response was identified, i.e. the feedback provided to the general practitioners. In a second stage, the threshold was again lowered to 15 % and the responses were grouped by proximity of this most contributive response (feedback to the GP; “fba*n*”), in Fig. 2.

#### Grouping case management projects with similar response patterns

Three types of projects could be identified, and they are grouped here by the type of response provided to each of the criteria. They are graded from one to three and can be seen as a continuum, going from “social” case management towards “clinical” case management.

A project of case management was assigned to a given type if it shared at least half of the response modalities of that particular type [32]. In total, 9 % were allocated to the Type 1 case management, 41 % to the Type 2 case management and 50 % to Type 3.

#### Type 1: social approach with low levels of collaboration and clinical components (right part of the graph in Fig. 2)

Type 1 is characterised by no feedback to the general practitioner of the beneficiary (*fba0*), no involvement of general practitioners and no indications that the projects’ stakeholders thought of it (*inc1*). Moreover, these projects are characterised by a very high turnover rate of the case (*tur1*) and reportedly insufficient adequacy of the inclusion criteria (*ade0*). However, these projects use evidence-based, multidisciplinary protocols (*ebp3*). The case manager teams in these types of projects do not include nurses nor social workers (*ICDC0*) but most often occupational therapists and/or psychologists. This led us to view this type of case management as a social strategy, in which the level of integration of the care poorly integrates the clinical components.

#### Type 2: social approach with integrative components (in the left corner of the same graph)

In Type 2, the level of feedback to the GP is still very low, as it only includes the information that the beneficiary benefits from case management (*fba1*). The care plan is poorly monitored (*mon1*) and there are no protocols available (*ebp0*). By contrast, these projects are able to recruit beneficiaries to whom case management would benefit the most (*ade3*); professionals delivering case management are adequately skilled (*ski2*), their training is adequately sustained by including external supervisors (*int3*) and the care provided is supported by software assisting the organisation of the care (*reg3*) but without prompts to providers (*rem0*). The team of case managers includes social workers and a psychologist or on occupational therapist (*IDC1*). In this type, the focus is on integration of the care at a social level. However, in comparison with the previous type, their intervention is likely to be more supported by the input of the other professionals, as they benefit from reflective discussions among peers including professionals from outside their organisation and the information about the beneficiaries is shared and organized through structured software. This type of social case management can be viewed as using strategies to strive towards integrated care through the use of high-skilled human resources and supervision, likely to delegate clinical components in adequate conditions.

#### Type 3: clinical integrative case management (centre of the graph)

This type of projects is characterized by the provision a high level of monitoring of the care plan (*mon3*) and whose professional profile of case manager is the most satisfactory, as they include experienced professionals whose training is supported by regularly planned reflective discussions among peers (*int2*), even if it is to a lesser level than in Type 2. Feedback to the beneficiary’s GP, including the results of the comprehensive assessment, is systematically provided (*fba3*). The work of the case managers is facilitated by the means of formal agreements with coordination centres (*par2*) and they can rely on shared software for the organisation of the care, allowing queries to sort beneficiaries by priorities and include specific information for the team about the results of the BelRAI (*reg2*). However, the inclusion criteria for the beneficiaries do not seem very adequate (*ade1*), possibly impairing the benefit the latter could experience from the project. The financial access to this type of case management can be impaired by the cost of the intervention, as older people have to pay more than 10€/day for the services recommended by the case managers (*fin1*). This led us to view these type of projects as clinical case management.

## Discussion

The evaluation of the effectiveness of complex interventions, such as case management, calls for innovative study designs [36]. The stepwise methodology, starting with the results of a case study analysis, identified 23 key components explaining the expected effectiveness of case management for community-dwelling frail older persons. This enabled the elaboration of a multimodal normative analysis grid, which can be used in evaluation research to make explicit important interacting components of newly implemented case management projects in primary healthcare systems and trigger discussion among stakeholders. The analysis continued with a multiple correspondence analysis (MCA). MCA was used to detect and explore relationships between structural and process components and offering statistical results that can be seen both analytically and visually. As in other studies [37–40], this study was able to illustrate the usefulness of multiple correspondence analysis (MCA) in detecting and representing underlying structures in a large dataset used to investigate key components likely to explain the effectiveness of case management for frail older people in Belgium.

This, in turn, enabled the grouping of case management types with similar response patterns. Findings are expected to have direct implications at the managerial and policy level, identifying case management programmes likely to have a desirable impact. We plan to carry out further research to test if these components are indeed related with better beneficiaries’ outcomes.

In this section, the typology of the case management programmes found in Belgium is presented and discussed in the views of the background literature. Secondly, key components of case management are listed. They are likely to generate positive clinical and social frail older people’s outcomes and confirm the relevance of Wagner’s Chronic Care model to guide the quality of the organisation of the care. Thirdly, limitations and strengths of our methodological design are outlined.

The visual representation of this quantitative analysis led to the distinction between three types of case management designs, in which the first type, acting in a social dimension, is marked by the poor level of collaboration with the beneficiaries’ GP. This is also associated with a high level of turnover rate of case managers, which are nor nurses, neither social workers. The inclusion criteria chosen are not seen as adequate while it is expected that case management will be more profitable to people deliberately identified as those with complex care needs [6, 41, 42]. The fact that this type of case management interventions use more often evidence-based, multidisciplinary protocols can maybe be seen as a compensation mechanism to counterbalance the lower skills of case managers regarding the complex care needed by this population [43]. An assumption regarding the high turnover rate is that this can both be a result and the cause of a low quality of case management. Indeed, at the one hand, if case managers perceive their care being of low quality they will also be dissatisfied by their job, in its turn linked with low retention rates. At the other hand, high turnover rates impede the building of the trusting professional relationships between the different care providers [44]. In the second type of case management, where the focus is also on the integration of the care at a social level, the intervention is likely to be more supported by the input of the other professionals, as they benefit from reflective discussions among peers including professionals from outside their organisation and the information about the beneficiaries is shared and organized thanks to a structured software. Therefore, communication flow between professionals about the beneficiaries’ complex health care needs is likely to be more fluent than in the previous group [45]. Eventually, in the third type of case management with a more clinical focus, the level of collaboration with other professionals is facilitated by external factors, such as formal agreements with other primary care agencies, the use of software for the organisation of the care, including sharing the information about the beneficiaries with other professionals. The collaboration is also facilitated by internal factors, such as the professional skills of the case managers, supported, by former experiences regarding geriatric care and ongoing reflective discussions among peers. The monitoring of the care plan, which in itself is supported by the means of evidence-based, multidisciplinary protocols is likely to provide positive results in this type of projects, as this enables an adequate view from the case manager on the possible instable situation of the beneficiary and propose tailored, evidence-based interventions. In its turn, this may lead to the prevention of acute exacerbations or, at least, attenuate their effects. This monitoring should also occur in other domains, such as monitoring of vital or clinical parameters (e.g. weight gain in kidney failure, glucose levels in diabetes), medication intake (e.g. chronic heart failure), depression symptoms, etc. It has to be stressed that the aforementioned monitoring cannot take place without the close involvement of the frail older people’s primary care physician, who should agree with what has to be monitored and when, in order to be able to link these functions of case management adequately with the other functions of case management. This means that the engagement of the primary care physician goes far beyond what was reported by most of the case management projects and may also be seen as a suboptimal way of providing case management in the observed projects [42]. The key components mentioned in these tree types confirm the usefulness and relevance of Wagner’s Chronic Care Model to guide the data collection and analysis for the evaluation of case management for frail older people.

Other authors have suggested typologies of case management interventions. One of the most cited is the critical review of Lee et al. [46] who describes the classification of Beardshaw and Towell [47, 48]. This classification makes the distinction between three models, [1] the *brokerage* model, in which advocacy is an important component and the case manager acts as an independent agent; [2] the *social entrepreneurship* model, where the case manager holds a budget for the purchase of care packages and [3] the *extension of the keyworker/care coordinator* function in which members of a multidisciplinary team deliver, coordinate and monitor the care provided. Models [1] and [3] appear to be close to Types 1 and 3 found in our study, i.e. social and clinical case management respectively; model 2 does not seem feasible in the Belgian context, where such care packages do not exist. Beardshaw and Towell stated that because of actual confusion about the structures and processes involved in the programmes, it was not possible to link any outcome to the impact of the case management. Fleisher’s review of “modern” case management models identified four categories of case management, based on dozen different models found in the literature. They are: the broker, the rehabilitation, the full support, and the strengths models. The *broker* model is similar to the brokerage model described by Lee et al. above [46] and to the Type 1 found in our own study, while the case manager in the *rehabilitation* model identifies strengths and deficits of the beneficiary and attempts to remedy a wide array of problems and barriers linked to his ability to function independently in the community. The *full support* model expands upon the rehabilitation model by using an integrated treatment team of providers and relies less on external referrals. This full support model is close our Type 3 model. In the *strengths* model, self-determination of the client and assisting the client in attaining client-specific goals are the core task of the case manager instead on basing his support on beneficiaries’ needs. This explains why the model puts a strong emphasis on the case manager-client relationship [48] A. The focus on self-determination was not made explicit in the projects in our study, which can be considered as a weakness of the case management approach for the population of older people. Indeed, loss of independency is too often assimilated with loss of autonomy [49]. Further, a common feature of the aforementioned classifications is that they do not link a specific type of case management to a given outcome or patient population. More recently, a systematic review of the AHRQ (2013) stated that because of the relatively low number of trials compare different types of case management models, conclusions about the features of programs that are most effective could be made only with a low strength of evidence [43]. One of the examples are some Dutch studies about different types of case management for older people with dementia and their informal caregivers [50]. Because of this, there is a need in future research to explicitly take into account the training received by case managers, the experiences and specific functions of case managers, the modes of contact (clinic visits, home visits, telephone calls), the average caseload, the relationship to other health care providers, the use of protocols, guidelines, and information technology ([43],p.16). In the grid we suggested, all these elements were taken into account, which opens the road to test the impact of these different types of case management interventions, for different types of populations, stratified for instance by level of complexity.

There are however some limitations to our study. Firstly, the sample limited to Belgian context calls for warnings about the transferability of the results of the grid and the typology coming out of the MCA in other countries. This weakness was countered by providing a rich description of the interaction of the components, allowing the reader to contextualise the information provided [24]. Secondly, warnings about the transferability of the results also apply because of the data collection including only the point of view of the stakeholders of the projects. Indeed, information bias might occur because this only reflects their view of the reality. Thirdly, although MCA allows the transformation of qualitative information into quantitative data to be used in further analysis, when qualitative variables are transformed into quantitative ones, valuable information may be lost [40]. Fourthly, the frontiers between the types of projects were fuzzy. The response modalities were provided in the close vicinity of the first response modality chosen, reflecting an *attraction* between these responses in the multiple component analysis. This means the typology resulting from the analysis does not represent an exclusivity of the response modalities. It is a simplification of the reality. In other words, projects attributed to a given type can show response modalities occurring in other types. The strength of this approach lies mainly in the synthesis of the data. This allows for the further testing of hypotheses, namely that clinical case management projects (Type 3 projects) will bring about better outcomes for beneficiaries with the most complex care needs. As for the use of the grid in other countries to evaluate the type of case management, the wording of the items of the grid may need refinement as to be applicable in other countries.

## Conclusion

Because case management programmes occur in complex and heterogeneous, multi-layered contexts in which they are embedded and with which they interact, the evaluation of the impact of these programmes needs to take into account these interactions. The novel study design proposed took into account these interactions and suggested a method to construct a typology of case management grouping case management programmes with similar interaction patterns. As such it is an important step to allow further impact evaluation, using these types for stratification while fostering discussion among stakeholders.

## Additional file

Additional file 1: Table S1. Criteria to evaluate the effectiveness and the implementation of the innovative case management projects.
