# Erratum to: A comprehensive grid to evaluate case management’s expected effectiveness for community-dwelling frail older people: results from a multiple, embedded case study

**DOI:** 10.1186/s12877-015-0084-2

**Published:** 2015-08-20

**Authors:** Thérèse Van Durme, Olivier Schmitz, Sophie Cès, Sibyl Anthierens, Roy Remmen, Patrick Maggi, Sam Delye, Johanna De Almeida Mello, Anja Declercq, Isabelle Aujoulat, Jean Macq

**Affiliations:** IRSS, Institute of Health and Society, Université catholique de Louvain clos Chapelle-aux-Champs, 30.13 B-1200 Brussels, Belgium; Faculty of Medicine and Health Care Sciences, Universiteit Antwerpen Universiteitsplein, 1 B-2610 Wilrijk, Belgium; Faculty of Public Health Université de Liège Avenue de l’hôpital, 3 B-4000 Liège, Belgium; LUCAS, Centre for Care Research and Consultancy KU Leuven (University of Leuven), Kapucijnenvoer 39, B-3000 Leuven, Belgium

## Erratum

The original version of this article [1] unfortunately contained a mistake. The sequence of the authors’ names was incorrect. The correct authors’ order is given below.

Thérèse Van Durme, Olivier Schmitz, Sophie Cès, Sibyl Anthierens, Roy Remmen, Patrick Maggi, Sam Delye, Johanna De Almeida Mello, Anja Declercq, Isabelle Aujoulat and Jean Macq
