# Supplementary material for: A comprehensive grid to evaluate case management’s expected effectiveness for community-dwelling frail older people: results from a multiple, embedded case study
Source: BMC Geriatr. 2015 Jun 18;15:67. doi: 10.1186/s12877-015-0069-1 (PMC4472179; doi:10.1186/s12877-015-0069-1)
Supplement: Additional file 1: Table S1. — Criteria to evaluate the effectiveness and the implementation of the innovative case management projects. [file 12877_2015_69_MOESM1_ESM.pdf]

Table 1. Criteria to evaluate the effectiveness and the implementation of the innovative case management projects

a. Appropriate workforce	
1. Recruitment of adequate professionals by projects	0 = could not recruit professionals
	1 = professionals with lower skills (training, expertise) were recruited
	2 = professionals with lower expertise were recruited
	3 = professionals with expected training and expertise were recruited
2. Skills of professionals	0 = no professional to provide this service
	1 = diploma of professional is not related to the service delivered
	2 = diploma of professional is related to the service delivered
	3 = diploma is related to the service delivered and this professional is experienced in this field
3. Turnover of professionals	0 = turnover of frontline worker and coordinator > once
	1 = turnover of frontline worker and coordinator = once
	2 = only frontline worker turnover
	3 = no turnover of professionals since start
4. Training of professionals	0 = no training planned
	1 = training planned, but could not organize it
	2 = internal training, not geriatric
	3 = internal training, specific to geriatric care
5. Number of frail older people per full-time equivalent case manager	0 = > 70/FTE case manager
	1 = 51-70/ FTE case manager
	2 = 41-50/FTE case manager
	3 = ≤ 40/FTE case manager
b. Tailored service design and organisation	
6. Achievement of the caseload	0 = achievement of <30% of the caseload
	1 = achievement of 31-50% of the caseload
	2 = achievement of 51-75% of the caseload
	3 = achievement of >75% of the caseload
7. Adequacy of the inclusion/exclusion criteria	0 = inclusion/exclusion criteria are not adequate and the project did not change them
	1 = inclusion/exclusion criteria were not adequate, the project changed them, but they are still inadequate

	3 = inclusion/exclusion criteria are adequate or were inadequate, but were adapted and are now adequate
8. Shared decision process	0 = no meetings including all the professionals of the project
	2 = professionals of the project attend meetings but do not share decision making/do not attend meetings of the steering group or do not receive minutes of those meetings
	3 = professionals of the project attend meetings and share decision making
c. Self-management and support	
9. Addressing concerns of beneficiaries and informal caregivers	0 = is not consistently done
	1 = percentage of informal caregivers OR frail older people attending a multidisciplinary meeting during last three months is LESS than median than this subgroup
	2 = percentage of informal caregivers OR frail older people attending a multidisciplinary meeting during last three months is MORE than median than this subgroup
	3 = percentage of informal caregivers AND frail older people attending a multidisciplinary meeting during last three months is MORE than median than this subgroup
d. Community linkages	
10. Existence of a structural link with organizations that can refer beneficiaries	0 = no link
	1 = convention signed, but no referral from beneficiaries
	2 = convention signed, referral less than expected
	3 = convention signed and referral as expected
11. Partnership with coordination agencies	0 = no link
	1 = ad hoc meetings, on initiative of project
	2 = ad hoc meetings, on initiative of coordination center
	3 = are reported through planned project meetings
12. Partnership with community organizations	0 = no link
	1 = are being considered, but not implemented
	2 = are part of the partnership
	3 = take part at project meetings
e. The appropriate financial incentives	
13. (Beneficiary view) financial access	0 = the beneficiary had to renounce services because of cost
	1= the beneficiary has to pay more than 10€/day for the service
	2 = the innovative intervention is free, but beneficiaries have to pay for the recommended services

	3 = the intervention is completely free for the beneficiary
14. Adequacy of the financing of the project	0 = the project intends to stop because it is not viable
	1 = the projects reports some financial difficulties and did not ask for extra financing from the NIHDI, or the financing was refused
	2 = the projects reports some financial difficulties and asked for extra financing from the NIHDI or frail older people, which was accepted
	3 = the project did not report any difficulties related to financing
15. Incentives for GP participation	0 = GPs are not involved and there are no indications that the project thought of it
	1 = GPs are not involved, the project suggested the GP to use of the nomenclature number related to multidisciplinary coordination, with no result
	2 = GPs are poorly involved, despite the financing provided by the project
	3 = GPs are well involved and financed by the project
f. Processes in support of quality of care	
16. Use of quality or performance indicators	0 = quality indicators are not mentioned
	1 = quality indicators are mentioned in submission files
	2 = monitoring of quality indicators is part of the discussion in steering committees meetings
	3 = results of quality indicators are provided
17. Monitoring of the care plan	0 = no individualized care plan
	1 = care plan only made once (e.g. at intake)
	3 = use and follow-up of care plan
18. Provision of feed-back of the frail older people to the GP	0 = no feedback provided
	1 = information of the frail older people being in the project
	2 = feedback provided, non structured
	3 = feed-back provided about the results of the BelRAI
g. Knowledge management and decision support	
19. Use of results of research	0 = the rationale on how the intervention will impact the frail older people outcomes is not mentioned or unclear
	1 = the rationale on how the intervention will impact the frail older people outcomes is based on projects' conceivers' perception
	2 = the rationale on how the intervention will impact the frail older people outcomes is based on results of research OR pilot study

	3 = the rationale on how the intervention will impact the frail older people outcomes is based on results of research AND pilot study
20. Use of evidence-based protocols or guidelines	0 = protocols or guidelines are not mentioned/not used
	1 = protocols or guidelines are mentioned in the submission files, but not later
	2 = protocols were made and/or used by the project and there is no information about their being linked to evidence-based literature
	3 = multidisciplinary protocols were made and used by the project and they rely on evidence-based literature
21. Presence of reflective discussions among peers/planned supervisions	0 = no such meetings are planned
	2 = meetings are planned and do not include a supervisor (physician or senior clinician)
	3=meetings are planned and include a supervisor
h. Clinical information tools	
22. Registry (list of beneficiaries of the projects)	0 = is not available
	1 = includes name, diagnosis, contact information and date of last contact either on paper or in a computer database
	2 = allows queries to sort beneficiaries by priorities
	3 = provides prompts and reminders about needed services
23. Reminders to providers	0 = are not available
	1 = include general notification of presence in the project, but do not describe the needed service
	3 = include specific information for the team about the results of the BelRAI
